# MarTurtSI, a global database of stable isotope analyses of marine turtles

**DOI:** 10.1038/s41597-019-0030-9

**Published:** 2019-04-03

**Authors:** Christine Figgener, Joseph Bernardo, Pamela T. Plotkin

**Affiliations:** 1 Marine Biology Interdisciplinary Program, Texas A&M University, College Station, TX 77843 USA; 2 Department of Biology, Texas A&M University, 3258 TAMU, College Station, TX 77843 USA; 3 Department of Oceanography, Texas A&M University, 3146 TAMU, College Station, TX 77843 USA; 4 Program in Ecology and Evolutionary Biology, Texas A&M University, College Station, TX 77843 USA; 5 Texas Sea Grant, Texas A&M University, 4115 TAMU, College Station, TX 77843 USA

**Keywords:** Animal migration, Herpetology, Animal behaviour, Stable isotope analysis, Behavioural ecology

## Abstract

Marine turtles are both flagship species of conservation concern and indicators of ocean health. As highly migratory species, and despite substantial research effort focusing on nesting females and satellite tagging studies, we still know little about the trophic ecology and habitat use of immature stages and males. Consequently, marine turtle biologists began using stable isotope analyses in the last decade to elucidate various aspects of trophic ecology, including habitat use and trophic position. This has resulted in a burgeoning but largely disconnected literature of mostly single-species case studies. Here we comprehensively synthesize this body of work into a unified data repository, the MarTurtSI database. MarTurtSI contains stable isotope data from six of seven marine turtle species ranging from juveniles to adults, in different developmental, feeding, and breeding habitats across multiple ocean basins. MarTurtSI will be curated and updated with the aim of enabling continued comprehensive and global investigations into the trophic ecology of marine turtles especially in the face of climate change and other conservation challenges.

## Abstract (summary)

**Table:** 

Design Type (s)	data integration objective • species comparison design
Measurement Type (s)	stable isotope analysis
Technology Type (s)	digital curation
Factor Type (s)	sex • geographic location • life cycle stage • Species
Sample Characteristic (s)	Chelonia mydas • skin of body • Pacific Ocean • ocean biome • Mediterranean Sea • Indian Ocean • Caretta caretta • muscle • scute • Atlantic Ocean • Lepidochelys olivacea • blood • Dermochelys coriacea • blood plasma • yolk • bone • Eretmochelys imbricata • Lepidochelys kempii

Machine-accessible metadata file describing the reported data (ISA-Tab format)

## Background & Summary

There are seven extant species of marine turtles and all are considered by the IUCN Red List of Threatened Species (http://www.iucnredlist.org, Version 2018-1) to be threatened at some level: the loggerhead (*Caretta caretta*, endangered), green (*Chelonia mydas*, endangered), leatherback (*Dermochelys coriacea*, vulnerable), hawksbill (*Eretmochelys imbricata*, critically endangered), flatback (*Natator depressus*, data deficient), Kemp’s ridley (*Lepidochelys kempii*, critically endangered), and olive ridley (*Lepidochelys olivacea*, vulnerable). Trophic ecology (habitat use, feeding niche) is crucial to understanding the biology of marine turtles. This is especially relevant because, like all highly migratory species, their management and conservation require both local and global efforts^1,2^. Furthermore, because marine turtles are at least tertiary consumers, except for herbivorous adult green turtles, their population dynamics must reflect changes in marine food webs, although this is not widely appreciated.

A long-standing limitation of marine turtle research, including stable isotope analyses, is an emphasis on localized data collection and lack of synthesis across studies. Stable isotope signatures provide insights into foraging habitat and trophic position but also serve as indicators of biogeochemical processes in the oceans^3,4^. Thus, a comprehensive database of stable isotope studies can provide insight into a variety of issues pertaining to marine turtles specifically and ocean health more generally. Data syntheses can inform conservation and management decisions, especially concerning habitat use by highly migratory turtles. Synthesis of diverse case studies also enables a search for emergent patterns in trophic ecology across regions and species, which has bearing on general ecological and evolutionary understanding of species coexistence. It also provides a baseline for future comparisons to facilitate the detection of changes in ecological processes among species and the oceans in the face of climate change.

**Fig. 1:**
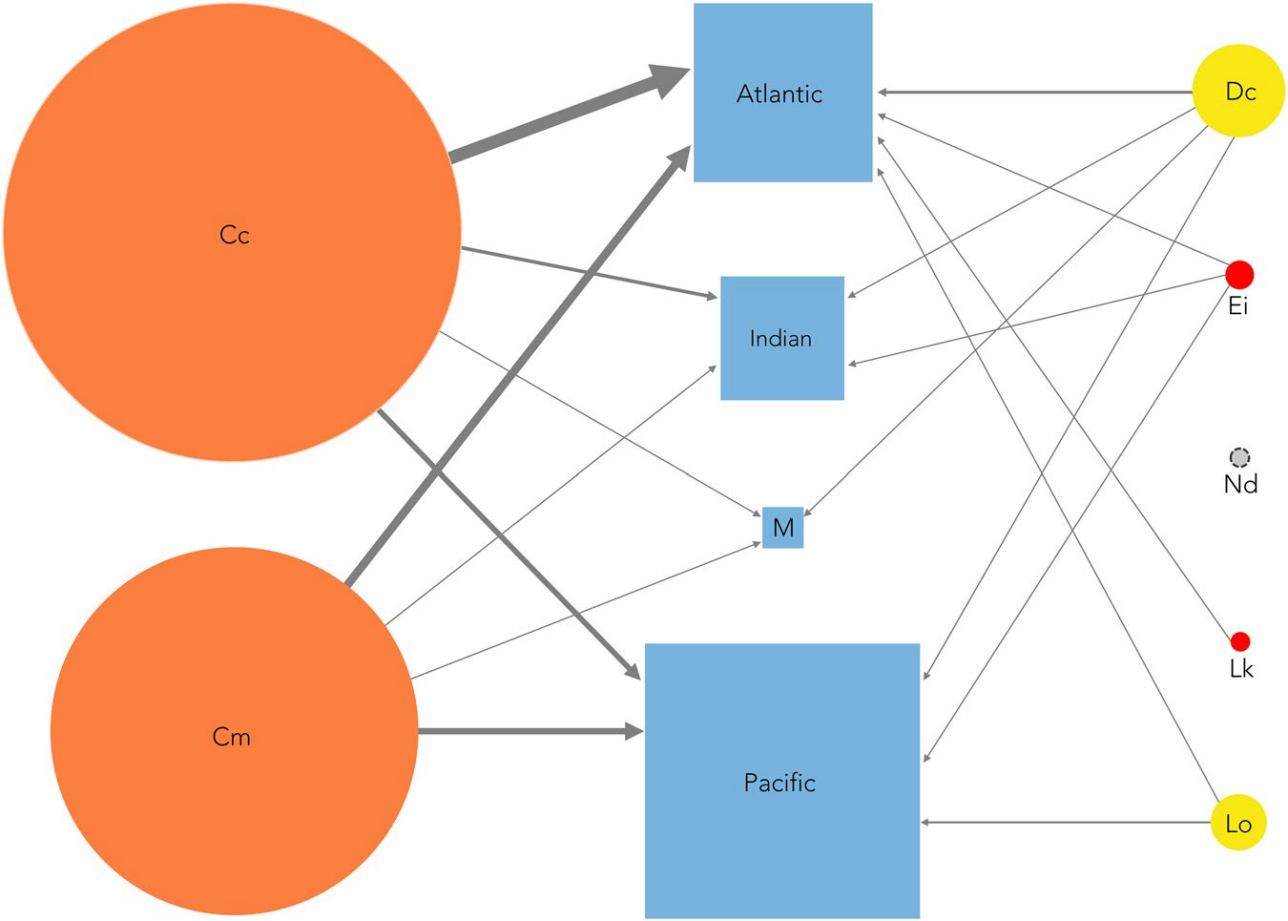
Graphical depiction of sampling density and distribution of stable isotope studies across marine turtles conveying seven dimensions of information. Circles represent the species (Cc = *Caretta caretta*, Cm = *Chelonia mydas*, Dc = *Dermochelys coriacea*, Ei = *Eretmochelys imbricata*, Nd = *Natator depressus*, Lk = *Lepidochelys kempii*, Lo = *Lepidochelys olivacea*). Circles are sized proportionally to the number of studies conducted per species, and are colored according to each species’ IUCN status (http://www.iucnredlist.org, Version 2018-1) using their color code (critically endangered = red, endangered = orange, vulnerable = yellow, data deficient = grey). We included a circle for *N*. *depressus* to represent the species and its threat status, but no studies have been conducted on this species. Squares represent ocean basins and are drawn proportionally to their area. We included the Mediterranean Sea (M) as a separate basin due to its limited connectivity to the Atlantic Ocean and to illustrate the large number of studies restricted to this basin. We did not include the Gulf of Mexico and the Caribbean Sea as separate basins due to their high connectivity to the rest of the Atlantic Ocean. Arrows represent studies of a particular species in a particular basin, and their width indicates the number of studies of each combination. This visual depiction of research effort immediately draws attention to several significant research gaps. The first is a taxonomic bias: half of all studies are conducted on *C*. *caretta* (49.6%) followed by *C*. *mydas* (39.8%), while the two most endangered species are poorly studied (*L*. *kempii*, 1.8%*; E*. *imbricata*, 2.7%*)*, and *N*. *depressus* is unstudied. The second is a geographic bias: most studies have been conducted in the Atlantic Ocean (60.2%), followed by the Pacific Ocean (35.4%). Only 10.5% of the studies were conducted in the Mediterranean Sea, and 4.4% in the Indian Ocean.

Here we present the MarTurtSI database comprising a list of all stable isotope studies found, a comprehensive data table with data from 113 studies of carbon and nitrogen isotopes in six of seven species, a subsidiary quantitative dataset with 91 records from 50 studies tabulating mean stable isotope values for adult marine turtles, and the raw data used to generate the subsidiary dataset. We extracted data from papers and classified the data using a set of type factors (*e*.*g*. species, ocean basins, life-stages, region, tissues analyzed). The resulting database thus synthesizes and summarizes the data in a form ready for researchers to use in broader analyses of the trophic ecology of marine turtles and other migratory marine species. Further, it provides a template for data reporting by future stable isotope studies to facilitate novel data comparisons among studies. Lastly, because the MarTurtSI database reveals both geographic and taxonomic bias in sampling of stable isotope signatures, it should stimulate future research aimed at closing gaps in our understanding of marine turtle biology and conservation (Fig. 1).

There are two other overviews of marine turtle stable isotope data^5,6^. The first^5^ focuses on identifying gaps in sampling effort for particular management units of conservation concern. It summarized the number of studies by species, management unit, IUCN category and tissue. In contrast to MarTurtSI, it did not extract quantitative estimates of stable isotope signatures, summarize specific research findings, nor did it detail and unify the different levels of heterogeneity in the data. The second^6^ comprises an online database of tabulated published data, including means and ranges, as reported in original papers. However, unlike MarTurtSI, it did not quantitatively extract raw data from figures to compute means, standard deviations, standard errors, and ranges. Also, that database only includes peer-reviewed studies and does not provide an overview and summary of key findings for each study. Hence, MarTurtSI differs substantially from both of these overviews.

## Methods

**Fig. 2:**
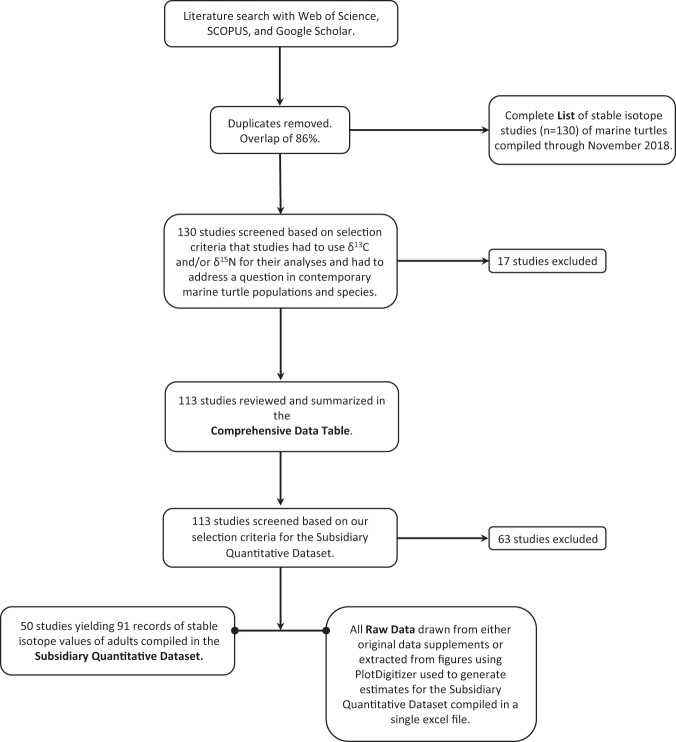
Flow chart of the process we used to generate and assemble the MarTurtSI database. It summarizes our search and screening procedures and the resulting documents included in MarTurtSI that we have produced^7^.

The process we undertook to assemble and generate the different components of MarTurtSI, a comprehensive database on trophic ecology in marine turtles, is summarized in Fig. 2. First, we conducted three independent literature searches using *Web of Knowledge*, *SCOPUS*, and *Google Scholar*. Keywords included but were not limited to: “sea turtle” and “marine turtle”, common and scientific names of the seven species, combined with “stable isotopes”, “foraging history”, “foraging ecology”, “trophic ecology”, or “trophic variation”. The results of these searches overlapped by 86%. Further, we examined literature cited sections of papers to identify additional studies not located through the search engines. Finally, we used citation alerts to find newly published papers. Most of the records were from peer-reviewed journal publications, but we also considered technical reports and unpublished theses if they included pertinent data that were not otherwise available. The initial data search yielded a list of 130 studies^7^, and the review process was concluded in November 2018.

Because our interest was in trophic ecology in contemporary species, the next step we took was to summarize these studies including only those using the stable isotopes δ^13^C and/or δ^15^N, which are the two most commonly used stable isotopes relevant to this question^8^ (Fig. 2). Thus, we excluded papers reporting data from other stable isotopes or from fossils. We included method papers relevant to understanding tissue turnover times and discrimination factors, as well as the effect of sample preparation and storage media on stable isotope signatures of δ^13^C and δ^15^N. This resulted in a final Comprehensive Data Table of 113 studies (Table 1). Each entry in the table includes isotopes reported and sample sizes and information for the classification factor types: species, ocean basin, region, life stage and tissues sampled. We also summarized the major findings of each study.

**Table 1:** Description of database MarTurtSI components with file locations (see Fig. 2 for method flow).

Source	Document Name	Number of Records (Studies)	Data Description	Methods
10.5061/dryad.3v060tq	List of Studies	130 (130)	Alphabetical list of references for stable isotope studies in marine turtles	Literature Search
10.5061/dryad.3v060tq	Comprehensive Data Table	113 (113)	Data table synthesizing studies of stable isotopes in marine turtles	Screening
10.5061/dryad.3v060tq	Subsidiary Quantitative Dataset	91 (50)	N, Mean, SD, SE of δ^13^C and δ^15^N, and body size of adult marine turtles	Data assimilation procedure
10.5061/dryad.3v060tq	Raw Data	40 Work Sheets	Raw data used to generate parts of Subsidiary Dataset.	Original supplementary materials from studies or extracted values with PlotDigitizer

Next, using the Comprehensive Data Table, we generated a dataset of quantitative estimates of δ^13^C and δ^15^N (Fig. 2), which could be analysed statistically to address our interest in marine turtle trophic ecology. In this Subsidiary Quantitative Dataset, we tabulated stable isotope means, standard deviation (SD), and/or standard error (SE), sample sizes, as well as the associated classification factor types described above. Additionally, we included data on body size (curved or straight carapace length and width) where available. We accepted means from studies of any tissue that reported the origin of samples, sample size, and either SD or SE within one nesting population or foraging area. We also accepted values from studies for which we could compute means and SE from either original supplementary datasets if available, or from datasets we generated by extracting values from published figures using PlotDigitizer 2.6.8 (http://plotdigitizer.sourceforge.net, 2001). The raw data that we used to compute the means and SE are available in the MarTurtSI Database^7^. If multiple estimates existed for the same species (different populations or different studies of the same population), we accepted all of them. We excluded certain studies that lacked sufficient detail (see Technical Validation below). The final Subsidiary Quantitative Dataset comprises values of δ^13^C and δ^15^N of adults from 50 of the 113 studies summarized in the Comprehensive Data Table. The dataset includes 91 records of values from six species (there were no studies of *N*. *depressus*), four ocean basins, and multiple tissues (Online-only Table 1).

## Data Records

The MarTurtSI database^7^ is available on *Dryad* and comprises four data files. The first is a table in MS Excel (.xlsx), which lists all studies found during the literature search, excluding duplicates (Table 1). The second file contains the summary of the reviewed stable isotope studies in marine turtles as a Comprehensive Data Table (Table 1) and is available in MS Word (.doc) and MS Excel (.xlsx) formats. The third file is the Subsidiary Quantitative Dataset, which contains stable isotope signatures of δ^13^C and δ^15^N for adult marine turtles. It is available in both MS Excel (.xlsx) and comma delimited (.csv) formats (Table 1). To facilitate future work, we also included a fourth file, an MS Excel spreadsheet, which tabulates all the raw data that we used to calculate entries for the Subsidiary Quantitative Dataset (Table 1) that were not directly reported in the published version of a study. Each tab pertains to a specific study and is either from the original supplementary data (modified to only include adults or in its original form (light blue tabs)) or the raw data extracted with PlotDigitizer (light green tabs).

Updates to the data and metadata of MarTurtSI will be curated through *Dryad* by creating a new version of the data package. We aim to include new studies and update all files in MarTurtSI annually.

## Technical Validation

There are two aspects of this work that yield technical validation of MarTurtSI. First, MarTurtSI reflects a high degree of marine turtle expertise both in the original works and in this data synthesis. All peer-reviewed studies and technical reports used as sources for this database were conducted or co-authored by marine turtle experts with substantial experience with both the organisms and techniques. The eight included theses were supervised by marine turtle experts. CF, who conducted the searches, extracted relevant information and assembled the database and PTP, who supervised this process, have 45 combined years of experience studying marine turtles (excepting *N*. *depressus*) in multiple ocean basins^9–16^.

Second, because differences among species, ocean basins, regions, life-stages, and tissues are known to cause variance in estimates of stable isotope signatures^3,4,17–20^, we defined classification factor types to unify and organize the data in a reproducible and statistically approachable way. These details were not always clearly articulated, and sometimes were absent from the original studies. We painstakingly extracted these details as part of our data compilation effort. For instance, some studies did not state the studied life-stage clearly and we had to determine it by examining their body size data. Hence, the data structure as captured in MarTurtSI will facilitate efforts by future researchers to partition the complex variation in stable isotope signatures^7^.

From the Comprehensive Data Table, we generated a Subsidiary Quantitative Dataset for analysis, which was restricted to adult turtles for two reasons. First, comparison of data from immature life-stages is notoriously variable owing to ontogenetic variation in diet, tissue-turnover times and discrimination factors related to growth rates^17,19,20^. Current data on juveniles and subadults are simply insufficient to tease this complexity apart (Online-only Table 1). By contrast, adult diets are relatively stable, and growth rates are negligible^21,22^, making comparisons among adult turtles tractable and valid. Second, of the four life-stages that have been studied, adult turtles represent the greatest species diversity enabling interspecific comparison (Online-only Table 1).

We rejected studies for which we were unable to obtain sample size, or an estimate of SD and/or SE, either because it was not reported or because we could not calculate them from data supplements or extracted data from figures. We also did not use data from studies that combined information from multiple populations or ocean basins in a single mean and that we could not disaggregate based on other information in the paper. A third reason we excluded some studies was that subgroup means were not reported with sample sizes.

## Usage Notes

**Fig. 3:**
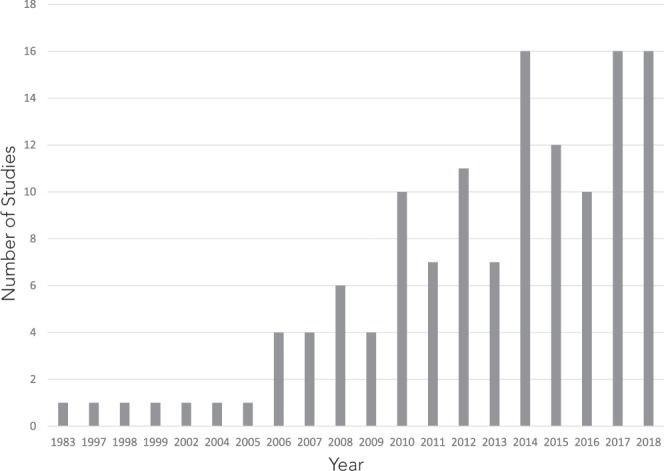
Illustration of the rapidly expanding literature on stable isotope studies of marine turtles from the first publication in 1983 until November 2018.

The literature on marine turtle stable isotopes has been growing rapidly (Fig. 3), a trend that is unlikely to abate. Prior to MarTurtSI, large-scale comparative analyses were hindered by the lack of uniformity in data reporting (*e*.*g*. not reporting sample sizes) and clear statement of classification factor types. MarTurtSI provides a data reporting template for future studies. This, in turn, should facilitate future comparative analyses of stable isotope heterogeneity at many hierarchical scales (*e*.*g*. comparing a given life-stage within a species across ocean basins; comparing different life-stages in the same ocean basin), shedding light on marine turtle trophic ecology from a wide variety of perspectives.

MarTurtSI also provides a clear picture of gaps in research effort with respect to species, endangerment status, and ocean basins that need to be addressed in future studies (Fig. 1). Hence, this synthesis should stimulate research to close these gaps.

### ISA-Tab metadata file

Download metadata file

## Online-only Tables

**Online-only Table 1:** Summary of species-specific research effort in marine turtle trophic ecology using stable isotope analysis (SIA) of δ^13^C and δ^15^N, partitioned separately by ocean basins, tissues used in SIA, and life-stages investigated. Some studies concern more than just one species, ocean basin, tissue, or life-stage.

	*Total*		Species							
			*Caretta caretta*	*Chelonia mydas*	*Dermochelys coriacea*	*Eretmochelys imbricata*	*Lepidochelys kempii*	*Lepidochelys olivacea*	*Natator depressus*	*Total*
			56	45	12	3	2	7	0	113
Ocean Basins	Atlantic Ocean including the Gulf of Mexico		33	21	7	3	2	2	*N/A*	68
	Mediterranean Sea		9	2	1	0	*N/A*	*N/A*	*N/A*	12
	Indian Ocean		1	2	1	1	*N/A*	0	0	5
	Pacific Ocean		15	16	3	1	*N/A*	5	0	40
	Captivity		1	3	1	0	0	0	0	5
Tissues	Blood	Plasma/Serum	5	6	2	0	0	1	0	14
		RBCs	7	5	3	0	0	1	0	16
		Whole	3	3	5	0	0	0	0	10
	Bone		14	6	2	0	0	1	0	23
	Egg	Albumen	3	0	0	0	0	0	0	3
		Shell	1	0	0	0	0	0	0	1
		Yolk	13	2	2	0	0	0	0	16
	Liver		0	0	1	0	0	0	0	1
	Muscle		4	4	1	0	1	0	0	10
	Offspring	Blood	1	0	0	0	0	0	0	1
		Liver	1	0	0	0	0	0	0	1
		Muscle	2	0	0	0	0	0	0	2
		Skin	1	0	0	0	0	0	0	1
	Scute		12	13	*N/A*	1	1	1	0	28
	Skin		24	26	6	2	0	5	0	63
Life-Stages	Hatchlings		4	0	1	0	0	0	0	5
	Juveniles		24	41	2	2	1	3	0	73
	Subadults		6	5	2	1	0	1	0	15
	Adults	Females	34	19	10	1	1	4	0	69
		Males	2	2	2	0	0	1	0	7
